# Increasing access to specialist care with group medical visits: summary of a pilot in a post-crisis psychiatric clinic

**DOI:** 10.3389/frhs.2023.1127725

**Published:** 2023-06-26

**Authors:** Josh Palay, James M. Bolton, Jitender Sareen, Jennifer M. Hensel

**Affiliations:** Department of Psychiatry, University of Manitoba, Winnipeg, MB, Canada

**Keywords:** group medical visit, shared medical appointment, access, mental disorder, pilot

## Abstract

**Background:**

Group medical visits (GMVs) have strong evidence of acceptability and effectiveness in the management of chronic medical diseases. Adaptation of GMVs for psychiatric care has potential to increase access, decrease stigma and save costs. Despite promise, this model has not been widely adopted.

**Methods:**

A novel GMV pilot was implemented for psychiatric care post-crisis among patients with primary mood or anxiety disorders who required medication management. Participants filled out PHQ-9 and GAD-7 scales at each visit in order to track their progress. After discharge, charts were reviewed for demographics, medication changes and symptom changes. Patient characteristics were compared between those who attended and those who didn't. Changes in total PHQ-9 and GAD-7 scores among attendees were assessed with paired *t*-tests.

**Results:**

Forty-eight patients were enrolled between October 2017 and the end of December 2018, 41 of whom consented to participate. Of those, 10 did not attend, 8 attended but did not complete, and 23 completed. Baseline PHQ-9 and GAD-7 scores did not differ significantly between groups. Significant and meaningful reductions in PHQ-9 and GAD-7 scores from baseline to last visit attended occurred among those who attended at least 1 visit (decrease of 5.13 and 5.26 points, respectively).

**Conclusions:**

This GMV pilot demonstrated feasibility of the model as well as positive outcomes for patients recruited in a post-crisis setting. This model has the potential to increase access to psychiatric care in the face of limited resources, however the failure of the pilot to sustain highlights challenges to be addressed in future pivots.

## Introduction

One of the greatest challenges for the future of mental health services is ensuring adequate access to specialist care among individuals with mental health disorders. This is a global issue with many jurisdictions reporting a gap in availability of psychiatrists in relation to the burden of societal mental illness (1, 2). This gap is even more pronounced among underserved and disenfranchised populations (3, 4). Numerous strategies have been proposed and implemented to optimize human health resource allocation, such as stepped care approaches, expanded scopes of practice of other behavioural health professionals, and increased indirect support for primary care such as embedded collaborative care and telephone or electronic consultation (5, 6). There still remains a high level of unmet need for specialist intervention among individuals who do not respond to initial treatments and exhibit refractory or complex illness.

One underused strategy to increase access to specialist treatment supervision is the group medical visit (GMV) or shared medical appointment. The GMV is a clinical encounter where patients with similar diagnoses attend shared medical appointments with the same health care provider(s) focusing on disease management (7, 8). In lieu of conventional one-on-one appointments, 2–8 (or more) patients can attend follow-up appointments together with a licensed health provider. Over the course of the appointment each patient is allotted time to discuss their own symptoms and treatment response and is given individualized recommendations. GMVs have a growing body of evidence supporting their acceptability and effectiveness for patients with chronic diseases such as diabetes, with many patients even preferring the group visit model to individual appointments (9).

A realist review of the published literature on GMVs identified 9 theoretical mechanisms of action through which GMVs work in particular contexts, supporting a rich foundation for their impact on practice and outcomes (7). In addition to benefit from improved access and efficiency of care delivery, GMVs offer many benefits of group dynamics and mutual learning opportunities for providers and patients (8). Moreover, GMVs have demonstrated the potential to reduce emergency department visits and hospital readmissions, while increasing problem-solving abilities and diagnosis-specific knowledge (10, 11). In a systematic review of GMVs in the primary care setting, this model was noted to contribute to gains in patient-centred outcomes including trust, perceived quality of care and quality of life, as well as clinical improvement (8).

Though there are numerous published evaluations of GMVs in obesity and diabetes care (12) and a range of non-diabetic chronic physical conditions (13), the literature on GMV adaptations to treat mental disorders is still lacking, with few examples available. Most studies examining GMVs for psychiatric patients come out of British Columbia, Canada where one program has widely adopted GMVs for routine outpatient psychiatric care (14). Results from that program have demonstrated patient satisfaction and potential cost-effectiveness (14, 15), and decreased feelings of mental health diagnosis-related stigma compared to individual treatment (16).

Despite growing evidence for their value, however, GMVs have not been widely adopted. Many authors have described barriers to optimal success including administrative and training factors, patient reluctance, and funding structures (17). An New England Journal of Medicine editorial published by Ramdas et al. in 2017 (18) highlighted the potential this service delivery model, and discussed 4 major barriers that have limited the widespread adoption of GMVs: lack of rigourous scientific evidence, easy ways to pilot and refine models, regulations and incentives, and patient and clinician education. The authors called for further investigation of GMVs, noting that the usage of “in-depth observational studies… to highlight subtle contextual variation will allow health systems and individual physicians to tailor shared appointments to specific patient populations” [(18), p. 1,106].

In this paper, we report on our own experience with a GMV in a post-crisis clinic where there was a need for ongoing psychiatric care among individuals with mood and anxiety disorders that wasn't feasible on an individual level with the available resources. We describe the approach to the delivery of the GMV in this novel population and the impact on patient outcomes. The goal of the pilot was to arrive at a feasible model that could be robustly evaluated and scaled. Drawing on the recommendations of Ramdas et al. (18), we highlight our own challenges with the operations and sustainability of the model and identify key factors necessary for future success.

## Materials and methods

### Setting and participants

Our clinic is located in a large urban centre in Winnipeg, Canada, situated within a mental health crisis facility offering 24/7 walk-in mental health assessment. Individuals presenting in crisis around the city to any of the 6 emergency departments and urgent care centres or to the 24/7 mental health crisis centre who do not require immediate psychiatric assessment or hospital admission may be referred to the post-crisis follow-up program. The follow-up program is managed by a multi-disciplinary team offering urgent psychiatric consultation (no follow-up), brief crisis counselling, and psychoeducation classes. Individuals referred for psychiatric consultation may be experiencing any kind of clinical presentation ranging from mood difficulties to psychosis and are usually offered an appointment within 2–4 weeks and recommendations are provided to the primary care provider. In general, access to urgent and ongoing psychiatric care is a major gap in our provincial health care system, resulting in care frequently being delivered in crisis settings and by primary care providers (PCPs) (19). While PCPs are usually comfortable with common and uncomplicated psychiatric presentations, they cite low confidence in managing more complex mental health issues (20). Because of the often-complex nature of psychiatric crises, and associated risk of deterioration or self-harm and suicide, many individuals seen in the post-crisis clinic would benefit from psychiatric follow-up and monitoring. Furthermore, a number of patients seen in our clinic do not have a PCP to whom recommendations may be made.

We implemented a GMV to provide follow-up psychiatric care for adults who received a post-crisis psychiatric consultation and (1) had a primary mood or anxiety disorder diagnosed, and (2) received treatment recommendations regarding medication management (start or change medications). Referred individuals were not required to have a PCP, in which case they were encouraged to secure one through a local primary care navigation service. If not accepting enrollment in the GMV, patients were advised to follow-up with their PCP per the usual standard of practice. All psychiatrists working in the post-crisis clinic were able to refer to the GMV. The number of psychiatrists varied over the course of the study but averaged between 2 and 3 with each offering 1–3 consultations in a given week. The exact volume of eligible individuals referred to the clinic is not known, but we would estimate approximately 30%–50% of those referred weekly receive a primary mood or anxiety disorder diagnosis.

We launched the GMV in October 2017 and enrolled individuals referred into the study until the end of 2018. Enrolled individuals were followed until the last one was discharged in July 2019. Additional individuals continued to referred to the GMV program in early 2019 but were not consented to participate in the study. GMV groups ran once a week throughout the duration of the study with the exception of provider vacations. A group was held provided at least 2 individuals were scheduled to attend.

### Approach to service design

Drawing on the recommendations from Ramdas et al. (18), we sought to pilot and refine our GMV for the target population using a flexible implementation approach that readily allowed adaptations based on early signals of benefit or lack thereof. In our GMV, visits were initially 60–75 min in length, occurred once a week, and accommodated 6–8 patients. They were run by a psychiatrist who had experience in group facilitation, with assistance from a psychiatry medical trainee or physician assistant. Administrative support was available for appointment scheduling and handling correspondence with PCPs. Program documentation included a combination of paper forms and electronic medical record entry. Each visit began with brief introductions, followed by each patient receiving approximately 5–10 min to discuss their individual progress. Symptom measures (Patient Health Questionnaire −9 item (PHQ-9) and the Generalized Anxiety Disorder Questionnaire-7 item (GAD-7)) were administered at every GMV to track progress and guide treatment. Measures such as weight and blood pressure were done when clinically indicated. In each session, *ad hoc* discussion and education were introduced based on the content and questions raised by patients. Individual enrollment in GMV was anticipated to be up to 12 weeks although this was not a hard endpoint, with discharge occurring once patients were stable on medication and suitable for routine follow-up with their PCP (or felt to need an alternate mental health program for longer term management). Attendance was not pre-set; that is, patients were invited to attend based on their preference and clinical need. As such, some individuals may have only required a single visit to review progress on the original recommendations whereas others may have attended weekly over the course of several months and could have received multiple medication trials. The goal of attendance was to achieve a meaningful reduction in symptoms and connect the individual with other services when required.

Based on feedback from patients and providers, early changes to the model included making the groups shorter (30–45 min), accommodating fewer patients per group (3–5), offering groups at 2 different times on the same day to accommodate more schedules, and allowing a longer duration of program attendance. Questionnaires were administered in the waiting room to reduce the time spent on this during the group.

Psychiatric care was reimbursed in a fee-for-service model during the implementation of the GMV and there was no specific billing code available for group psychiatric care. As such, a modified billing structure was adopted consisting of a combination of individual psychiatric care and group psychotherapy.

### Data collection

Individuals accepting referral to the GMV provided concealed consent to use their participation data for evaluation purposes. They reviewed a document summarizing how their data would be used, and then opted in or out by signing the consent and placing it in a sealed envelope that was not opened until they were discharged from the program. After an individual was discharged, the consent was reviewed and data on baseline demographics, attendance (number of sessions, weeks in program), and scores on the PHQ-9 and GAD-7 from enrollment to discharge were retrospectively extracted by chart review. Total scores were calculated respectively as the sum of the 7 items on the GAD-7 and the 9 items on the PHQ-9. Both scales use a Likert response scale ranging from 0-Not at all to 3-Nearly every day. Scores of 10 or more indicate potentially concerning levels of anxiety or depression. Measured longitudinally, changes of 5 or greater in PHQ-9 total score reflect clinically important differences in individuals receiving depression treatment (21), and 4 or more in GAD-7 for psychiatric patients receiving anxiety treatment (22). Approximately quarterly throughout the delivery of the pilot, the clinical team documented what was working well and where challenges existed to inform service planning decisions. These notes were reviewed with the team at study close to summarize the barriers to the pilot's success.

### Ethics

This study was approved by the University of Manitoba Research Ethics Board and the Winnipeg Regional Health Authority Research Access & Approval Committee.

### Data analysis

Demographic and clinical characteristics of individuals referred to the GMV program were summarized, stratified by whether or not they attended. We plotted mean total scores on the PHQ-9 and GAD-7 at baseline and discharge (last visit) for the group of individuals who attended and completed the program, and for those who started but did not complete. We also plotted a baseline score for those who never attended and compared baseline symptom severity between these groups with Analysis of Variance. Change in PHQ-9 and GAD-7 measures from baseline to discharge was assessed for GMV attendees, both completers and non-completers combined, with a general linear model, including time as a repeated measure and number of GMVs attended as a covariate.

## Results

**Figure 1:**
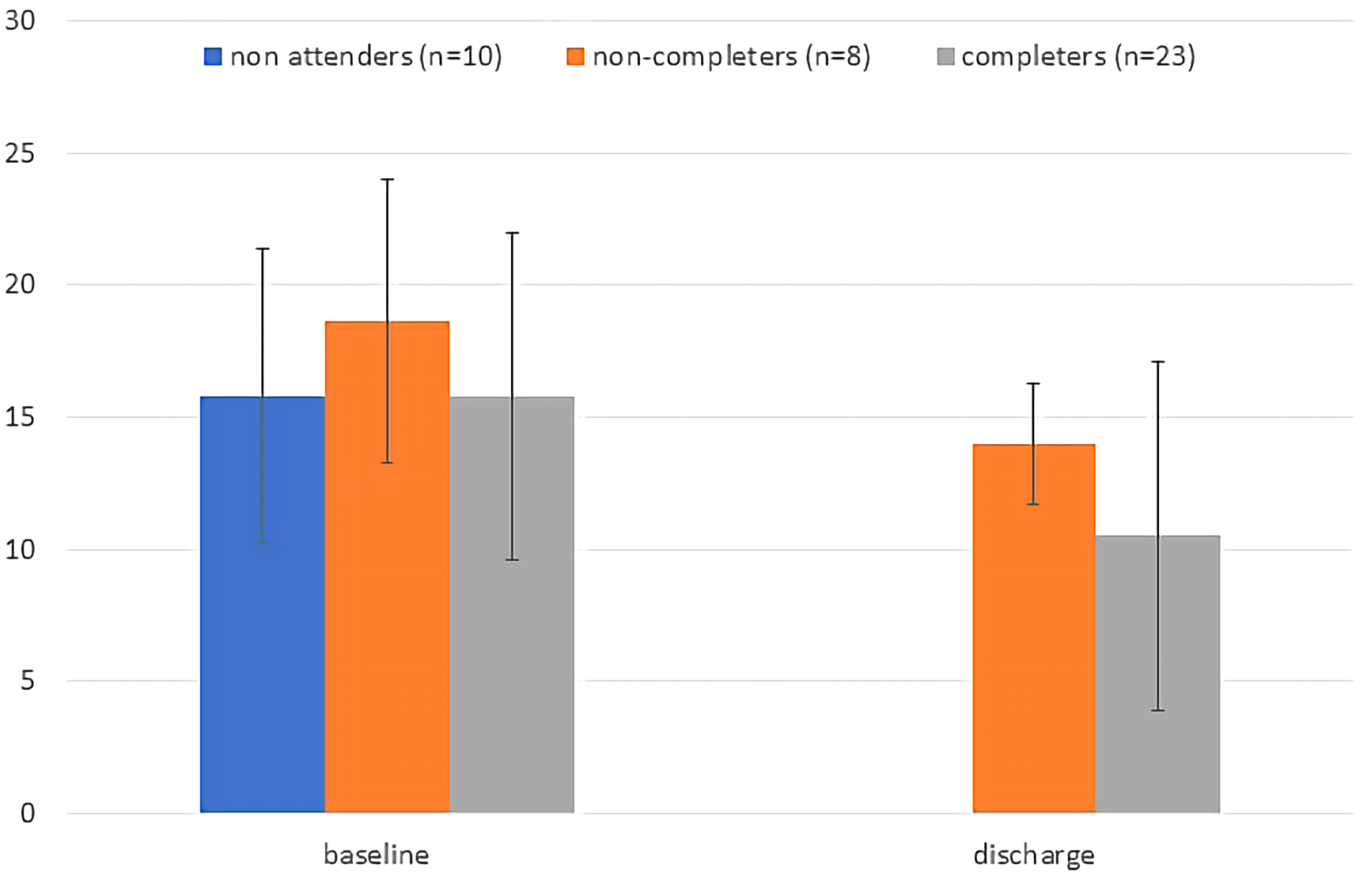
Baseline and discharge total PHQ-9 scores plotted by group. For non-completers discharge score is the score at last visit attended. Error bars are standard deviations.

**Figure 2:**
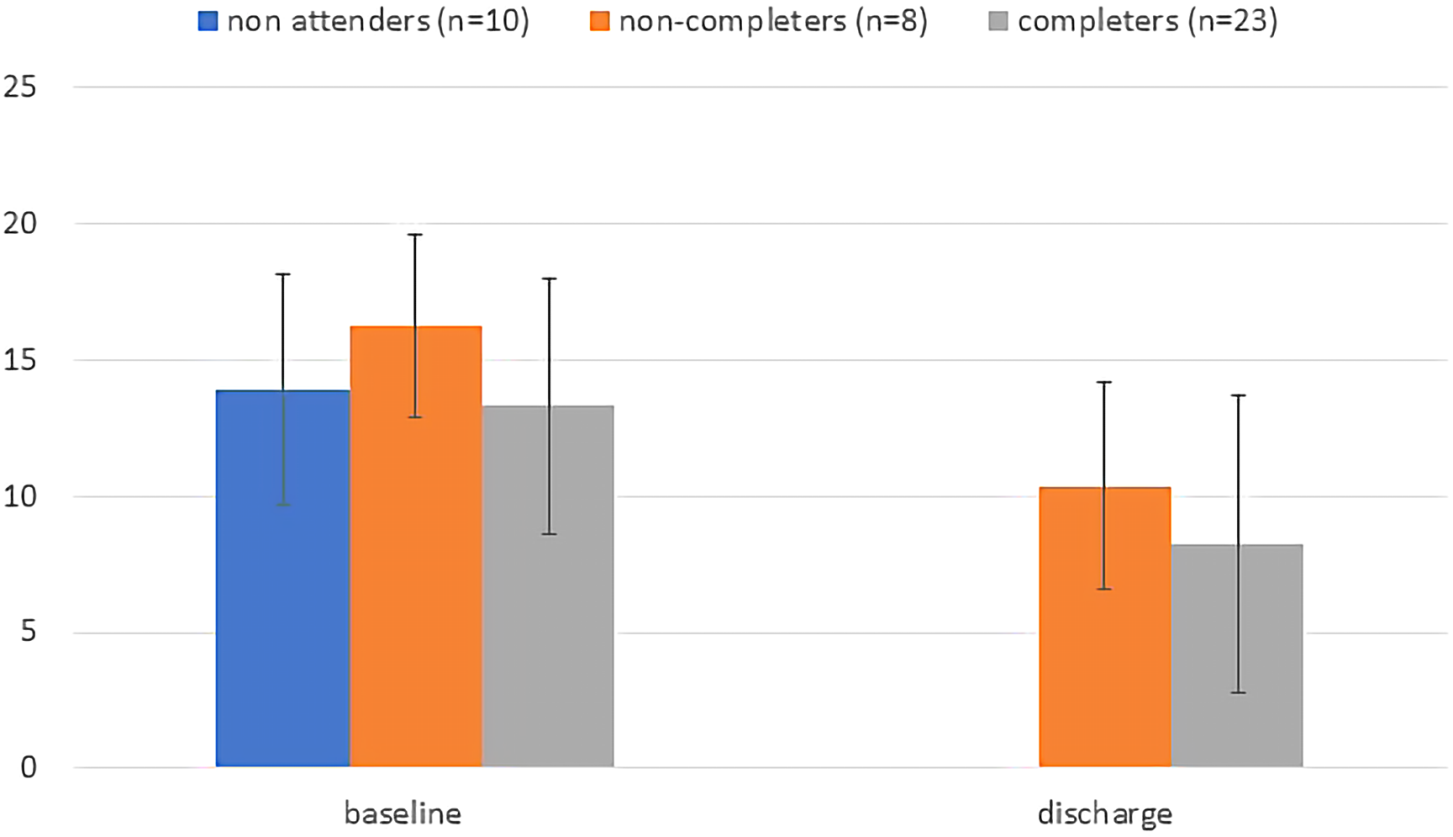
Baseline and discharge total GAD-7 scores plotted by group. For non-completers discharge score is the score at last visit attended. Error bars are standard deviations.

Forty-eight individuals were enrolled in GMV, 41 (85.4%) of whom consented to the use of their data for evaluation purposes (2 declined, 5 did not complete it). Ten of those 41 individuals (24.4%) did not attend a single GMV. Characteristics of those who attended and did not are found in Table 1. The 31 active participants attended a median of 5 visits (IQR: 3–10), over a median of 14 weeks (IQR: 5–33). Eight (25.8%) individuals were lost to follow-up (did not complete); 23 (74.2%) were formally discharged (completed), 17 of them to primary care, and 2 each (6 total) to individual psychiatric care, other mental health services, or hospital. Baseline and discharge PHQ-9 and GAD-7 scores are shown in Figures 1, 2, respectively (last visit scores are shown for those who attended but did not complete). Baseline PHQ-9 and GAD-7 scores did not differ significantly between those who never attended, those who attended but did not complete, and those who completed the program [F(2,38) = .73, *P* = .489 and F(2,38) = 1.36, *P* = .269, for PHQ-9 and GAD-7 respectively]. For individuals attending at least 1 GMV, regardless of whether they completed or not, there was a significant effect of time on PHQ-9 [F(1,29) = 12.1, mean decrease of 5.13, *P* = .001] and GAD-7 [F(1,29) = 16.7, mean decrease of 5.26, *P* < .001] scores, indicating improvement from baseline to last visit attended. The interaction between time and number of GMVs attended was not significant for either PHQ-9 [F(1,29) = 2.68, *P* = .133] or GAD-7 [F(1,29) = 1.75, *P* = .196]. Medication augmentation strategies were evident at discharge; higher rates of atypical antipsychotics and mood stabilizers were being prescribed.

**Table 1:** Demographics, psychiatric history, and medications (baseline and discharge) for patients referred to the GMV, stratified by whether they attended at least one GMV.

	Patients who attended GMVs (*n* = 31)	Patients who did not attend GMVs (*n* = 10)
Age, mean (SD)	37.7 (12.9)	29.2 (9.3)
Male gender, *n* (%)	9 (31)	0 (0)
Unemployed or off work, *n* (%)	17 (55)	3 (33)
**Primary mental health diagnosis, *n* (%)**		
Depressive disorder	27 (87)	6 (60)
Generalized Anxiety Disoder	0 (0)	2 (20)
Other Anxiety Disorder	2 (7)	1 (10)
Bipolar Disorder	2 (7)	1 (10)
Comorbid mental health diagnoses, *n* (%)	26 (84)	6 (60)
Comorbid active substance use, *n* (%)	4 (13)	2 (20)
Lifetime/Past-Year hospitalization, *n* (%)	6 (21)/2 (7)	1 (11)/0 (0)
Lifetime/Past-year suicide attempt, *n* (%)	9 (31)/2 (7)	2 (22)/1 (11)
**Medications at baseline, *n* (%)** ^a^		
None	4 (14)	4 (40)
SSRI or SNRI	19 (61)	6 (60)
Other Antidepressant	8 (26)	–
Lithium	1 (3)	–
Other Mood Stabilizer	2 (7)	–
Antipsychotic	5 (17)	1 (10)
Benzodiazepine	9 (29)	2 (20)
**Medications at discharge, *n* (%)** ^a^ ^,^ ^c^		
SSRI or SNRI	28 (90)	–
Other Antidepressant	9 (29)	–
Lithium	2 (7)	–
Other Mood Stabilizer	3 (10)	–
Antipsychotic	10 (32)	–
Other Psychiatric^b^	2 (7)	–
Benzodiazepine	9 (29)	–

^a^ Not mutually exclusive.

^b^ Prazosin.

^c^ Discharge is last recorded visit.

From the perspective of the clinical team, the most significant challenges to the delivery of the GMV were: (1) achieving adequate referral volumes given the variability in referrals to the clinic and inter-psychiatrist practice variability, (2) high patient acuity and few outflow options, and (3) inadequate administrative and other personnel support. There was a high turnover of both administrative and clinical support staff, at times without consistent coverage. As a result of these challenges, groups were often under-attended and run by a solo practitioner. After roughly 1.5 years, the pilot GMV was discontinued with goals to implement in a different setting with higher patient volumes and more clinical support. The onset of the COVID-19 pandemic and a shift to virtual care resulted in this being put on hold.

## Discussion

Our experience with a GMV pilot for individuals accessing psychiatric care through a post-crisis clinic demonstrated feasibility and acceptability of the model as well as positive outcomes for patients, but also highlighted significant challenges to be addressed in order for the model to be sustained. Notably, the average improvements seen among attendees in both the PHQ-9 and GAD-7 achieved statistical significance and exceeded the accepted cutoff for meaningful clinical improvement (21, 22). While we have demonstrated the potential benefit of GMVs for symptom reduction among patients in our post-crisis psychiatry clinic, our analysis is limited due to the small sample size, the variability in the timing of the outcome measures, and the lack of a comparison group. That said, the goal of this pilot was to implement and refine a service before we attempted to undertake a large-scale evaluation. The changes made allowed us to accommodate patient preferences; however, we did not examine whether this affected patient acceptability of the model or actual attendance. There was strong support from leadership and protected time for model development and implementation which were positive aspects of the setting and noted to be facilitators of success in other GMV programs. However, adequate personnel and administrative support were not available. In addition, the fee-for-service payment structure did not majorly incentivize the new model of care, such that the added workload was not well balanced with the remuneration. These limitations, paired with insufficient patient volumes and patients who often needed extended care in the absence of available outflow options, ultimately resulted in discontinuation of the program and the pursuit of further evaluation.

Both the potential benefits seen as well as the challenges we encountered have been described by others. Administrative and resource challenges were the most commonly cited barriers identified in a systematic review by Graham et al. (17). This category included co-ordination tasks as well as funding, and limited staff availability. Graham et al. (17) also identified barriers related to patient resistance and suitability with providers noting some patients didn't want to show up and had skepticism about the group model compared to usual 1:1 care. We also saw a portion of individuals who accepted enrollment but never attended suggesting that they may not have been interested in receiving care in the group format. Additionally, we do not know what proportion of potentially eligible individuals declined the invitation to participate altogether as we did not have the resources to track this. Wong et al. (23) have discussed why some individuals may not identify with the group format as a preferred form of receiving care, while others will prefer it.

To achieve the objective of increasing access to specialist care, the GMV has to provide access for more individuals with the same amount of provider time (or less) and result in similar outcomes. In order to accomplish this, there needs to be good support for the provider and processes. Much of the GMV could be automated with digital questionnaires and appointment booking. On a similar note, a number of groups have described the adaptation of the GMV to be conducted over videoconference prior to and during the COVID-19 pandemic (24, 25). There is also some evidence to support that GMVs can have significant benefit on the provider in terms of having extended time with patients, and team-based care (26). As others have suggested (18, 27), there is a need for more rigorous evaluation of GMVs across diverse populations and conditions and for clinicians to report on their successes and failures so the rest of the clinical community can learn from it. Policy makers and planners could aim to consider how GMVs fit within the overall local health care system to outline necessary pathways and resources for their optimization. For example, Jones et al. discuss a systems approach to implementing GMVs in the United Kingdom's National Health System (28).

Locally, we have not yet launched a second iteration of GMVs in our mental health program but we continue to discuss the possibility within various services including a high-volume general consultation service as well as in specialized longer-term treatment programs. In the next iteration, strong administrative support, a team-based approach where psychiatrists work with other mental health professionals like nurses and physician assistants, and determination of a competitive fee structure to incentivize clinicians working in a fee-for-service payment model will need to be obtained. For complex patient populations such as the target of this pilot GMV, group medical support may not be sufficient on its own; a combined approach where this is one facet of their care could support enhanced recovery and optimize scarce psychiatrist resources. The fact that our pilot GMV failed to sustain provides invaluable learning to inform a pivot to a new setting where the model may be a better fit. Additionally, longer term evaluation, ideally with a comparison group, is needed to further assess the impact of this care model on outcomes in psychiatric settings including clinical improvement, quality of life and recovery, and health economic benefit. This is a model with great potential that could be useful in addressing the existing treatment gap for psychiatric illness.

## Data Availability

The raw data supporting the conclusions of this article will be made available by the authors, without undue reservation.

## References

[1] Satiani A Niedermier J Satiani B Svendsen DP. Projected workforce of psychiatrists in the United States: a population analysis. Psychiatr Serv. (2018) 69(6):710–3. 10.1176/appi.ps.201700344 29540118

[2] Butryn T Bryant L Marchionni C Sholevar F. The shortage of psychiatrists and other mental health providers: causes, current state, and potential solutions. Int J Acad Med. (2017) 3:5–9. 10.4103/IJAM.IJAM_49_17

[3] Mongelli F Georgakopoulos P Pato MT. Challenges and opportunities to meet the mental health needs of underserved and disenfranchised populations in the United States. Focus. (2020) 18(1):16–24. 10.1176/appi.focus.20190028 32047393 PMC7011222

[4] Roberts T Miguel Esponda G Torre C Pillai P Cohen A Burgess RA. Reconceptualising the treatment gap for common mental disorders: a fork in the road for global mental health? Br J Psychiatry. (2022) 221(3):553–7. 10.1192/bjp.2021.221 35137680

[5] Raviola G Naslund JA Smith SL Patel V. Innovative models in mental health delivery systems: task sharing care with non-specialist providers to close the mental health treatment gap. Curr Psychiatry Rep. (2019) 21(6):44. 10.1007/s11920-019-1028-x 31041554

[6] Shidhaye R Lund C Chisholm D. Closing the treatment gap for mental, neurological and substance use disorders by strengthening existing health care platforms: strategies for delivery and integration of evidence-based interventions. Int J Ment Health Syst. (2015) 9:40. 10.1186/s13033-015-0031-9 26719762 PMC4696279

[7] Kirsh SR Aron DC Johnson KD Santurri LE Stevenson LD Jones KR A realist review of shared medical appointments: how, for whom, and under what circumstances do they work? BMC Health Serv Res. (2017) 17(1):113. 10.1186/s12913-017-2064-z 28160771 PMC5291948

[8] Wadsworth KH Archibald TG Payne AE Cleary AK Haney BL Hoverman AS. Shared medical appointments and patient-centered experience: a mixed-methods systematic review. BMC Fam Pract. (2019) 20(1):97. 10.1186/s12875-019-0972-1 31286876 PMC6615093

[9] Housden L Wong ST Dawes M. Effectiveness of group medical visits for improving diabetes care: a systematic review and meta-analysis. Cmaj. (2013) 185(13):E635–44. 10.1503/cmaj.130053 23939218 PMC3778483

[10] Trento M Passera P Borgo E Tomalino M Bajardi M Cavallo F A 5-year randomized controlled study of learning, problem solving ability, and quality of life modifications in people with type 2 diabetes managed by group care. Diabetes Care. (2004) 27(3):670–5. 10.2337/diacare.27.3.670 14988283

[11] Beck A Scott J Williams P Robertson B Jackson D Gade G A randomized trial of group outpatient visits for chronically ill older HMO members: the cooperative health care clinic. J Am Geriatr Soc. (1997) 45(5):543–9. 10.1111/j.1532-5415.1997.tb03085.x 9158573

[12] Trickett KH Matiaco PM Jones K Howlett B Early KB. Effectiveness of shared medical appointments targeting the triple aim among patients with overweight, obesity, or diabetes. J Am Osteopath Assoc. (2016) 116(12):780–7. 10.7556/jaoa.2016.153 27893144

[13] Kelly F Liska C Morash R Hu J Carroll SL Shorr R Shared medical appointments for patients with a nondiabetic physical chronic illness: a systematic review. Chronic Illn. (2019) 15(1):3–26. 10.1177/1742395317731608 28927284

[14] Remick RA Araki Y Bruce R Gorman C Allen J Remick AK The mood disorders association of British Columbia psychiatric urgent care program: a preliminary evaluation of a suggested alternative model of outpatient psychiatric care. Can J Psychiatry. (2014) 59(4):220–7. 10.1177/070674371405900407 25007115 PMC4079133

[15] Adams DJ Remick RA Davis JC Vazirian S Khan KM. Exercise as medicine-the use of group medical visits to promote physical activity and treat chronic moderate depression: a preliminary 14-week pre-post study. BMJ Open Sport Exerc Med. (2015) 1(1):e000036. 10.1136/bmjsem-2015-000036 27900130 PMC5117054

[16] Remick RA Remick AK. Do patients really prefer individual outpatient follow-up visits, compared with group medical visits? Can J Psychiatry. (2014) 59(1):50–3. 10.1177/070674371405900109 24444324 PMC4079228

[17] Graham F Tang MY Jackson K Martin H O’Donnell A Ogunbayo O Barriers and facilitators to implementation of shared medical appointments in primary care for the management of long-term conditions: a systematic review and synthesis of qualitative studies. BMJ Open. (2021) 11(8):e046842. 10.1136/bmjopen-2020-046842 34429309 PMC8386233

[18] Ramdas K Darzi A. Adopting innovations in care delivery - the case of shared medical appointments. N Engl J Med. (2017) 376(12):1105–7. 10.1056/NEJMp1612803 28328325

[19] Rush B Roberts A Cheung A Furlong A Ramirez J Butt P Improving access and coordination of mental health and addiction services: a provincial strategy for all manitobans. *Winnipeg, Manitoba: Virgo Planning and Evaluation Consultants Inc*. (2018). Available at: https://www.gov.mb.ca/mh/mh/docs/mha_strategic_plan.pdf. (Accessed June 19, 2023).

[20] http://ontario.cmha.ca/public_policy/opening-doors.

[21] Löwe B Unützer J Callahan CM Perkins AJ Kroenke K. Monitoring depression treatment outcomes with the patient health questionnaire-9. Med Care. (2004) 42(12):1194–201. 10.1097/00005650-200412000-00006 15550799

[22] Beard C Björgvinsson T. Beyond generalized anxiety disorder: psychometric properties of the GAD-7 in a heterogeneous psychiatric sample. J Anxiety Disord. (2014) 28(6):547–52. 10.1016/j.janxdis.2014.06.002 24983795

[23] Wong ST Lavoie JG Browne AJ MacLeod ML Chongo M. Patient confidentiality within the context of group medical visits: is there cause for concern? Health Expect. (2015) 18(5):727–39. 10.1111/hex.12156 24314271 PMC5060870

[24] Mirsky JB Thorndike AN. Virtual group visits: hope for improving chronic disease management in primary care during and after the COVID-19 pandemic. Am J Health Promot. (2021) 35(7):904–7. 10.1177/08901171211012543 33906426

[25] Tokuda L Lorenzo L Theriault A Taveira TH Marquis L Head H The utilization of video-conference shared medical appointments in rural diabetes care. Int J Med Inform. (2016) 93:34–41. 10.1016/j.ijmedinf.2016.05.007 27435945

[26] Thompson-Lastad A Gardiner P. Group medical visits and clinician wellbeing. Glob Adv Health Med. (2020) 9:2164956120973979. 10.1177/2164956120973979 33282545 PMC7683834

[27] Barnett KG. Group medical visits: the future of healthcare? Glob Adv Health Med. (2015) 4(6):6–7. 10.7453/gahmj.2015.110 26665014 PMC4653600

[28] Jones T Darzi A Egger G Ickovics J Noffsinger E Ramdas K, et al. Process and systems: a systems approach to embedding group consultations in the NHS. Future Healthc J. 6. 2019. 8–16. 10.7861/futurehosp.6-1-8 31098579 PMC6520080

